# Fluorinated Organic Paramagnetic Building Blocks for Cross-Coupling Reactions

**DOI:** 10.3390/molecules25225427

**Published:** 2020-11-19

**Authors:** Larisa V. Politanskaya, Pavel A. Fedyushin, Tatyana V. Rybalova, Artem S. Bogomyakov, Nargiz B. Asanbaeva, Evgeny V. Tretyakov

**Affiliations:** 1 N.N. Vorozhtsov Novosibirsk Institute of Organic Chemistry, 9 Ac. Lavrentiev Avenue, 630090 Novosibirsk, Russia; plv@nioch.nsc.ru (L.V.P.); feduyshin@nioch.nsc.ru (P.A.F.); rybalova@nioch.nsc.ru (T.V.R.); nasanbaeva@nioch.nsc.ru (N.B.A.); 2 International Tomography Center, Siberian Branch of Russian Academy of Sciences, 3a Institutskaya Str., 630090 Novosibirsk, Russia; bus@tomo.nsc.ru; 3 N.D. Zelinsky Institute of Organic Chemistry, Leninsky Prospect, 47, 119991 Moscow, Russia

**Keywords:** stable organic radicals, functionalized nitroxides, *tert*-butylarylnitroxides, fluoroarenes, aromatic nucleophilic substitution

## Abstract

New stable polyfluorinated nitroxide radicals for use in cross-coupling reactions, namely, *N*-*tert*-butyl-*N*-oxyamino-2,3,5,6-tetrafluoro-4-iodobenzene and *N*-*tert*-butyl-*N*-oxyamino-2,3,5,6-tetrafluoro-4-ethynylbenzene, were prepared from perfluoroiodobenzene. The reaction of the polyfluoro derivative with *tert*-butylamine under autoclaving conditions leading to the formation of *N*-*tert*-butyl-2,3,5,6-tetrafluoro-4-iodoaniline proved to be the key stage of the whole process. The fluorinated *tert*-butyl iodophenyl nitroxide was found to form in a solid state via N–O···I halogen bonds, a one-dimensional assembly of the radicals. The acceptor role of the nitroxide group in the halogen bonding changes to a donor role when the nitroxide reacts with Cu(hfac)_2_. In the last case, zero-dimensional assembly prevails, giving a three-spin complex with axial coordinated nitroxide groups and, as a consequence, causing ferromagnetic intramolecular exchange interactions between Cu(II) and radical spins.

## 1. Introduction

*tert*-Butylarylnitroxides and corresponding polynitroxides are an important class of paramagnets that have been actively used in different fields of research. A typical method for the preparation of *tert*-butylarylnitroxides involves an interaction of an appropriate aromatic organometallic compound with *tert*-nitrosobutane, thus giving aryl *tert*-butylhydroxylamine, followed by its oxidation to the target radical product. Less commonly, the oxidation of *N*-*tert*-butylanilines is performed to obtain nitroxide radicals [1,2].

**Figure 1:**
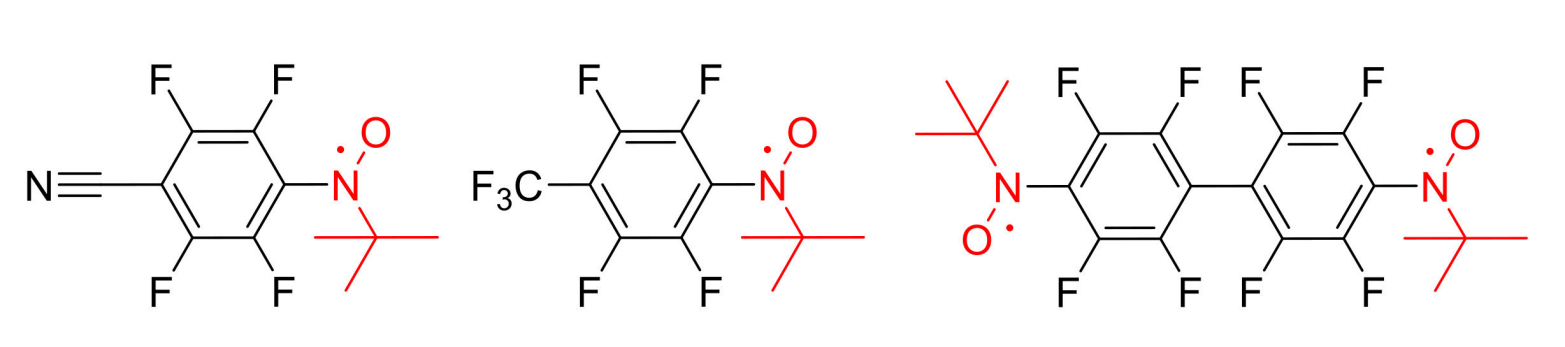
Synthesized polyfluorinated *tert*-butylarylnitroxides.

Recently, we proposed a new synthetic approach to obtaining functionalized *tert*-butylphenylnitroxides by sequential substitution of a fluorine atom in polyfluorinated arenes with *tert*-butylamine or lithium *tert*-butylamide and oxidation of resultant *N*-*tert*-butylanilines with *meta*-chloroperoxybenzoic acid (*m*-CPBA). This approach has been found to generate nitroxide mono- and diradicals in high yields (Figure 1) [3,4]. Moreover, it turned out that polyfluorinated *tert*-butylarylnitroxides are much stable than nonfluorinated analogs. Therefore, the nitroxides of this type can serve as paramagnetic building blocks for diverse applications.

In particular, one of the tasks of our current project aimed at obtaining spin-labeled graphene nanostructures is to search for stable paramagnetic blocks suitable for introduction into polycondensed aromatic structures and capable of efficiently injecting spin density into such structures [5,6,7,8]. Because the above-mentioned polyfluorinated *tert*-butylarylnitroxides satisfy all these requirements, within the framework of this work, we studied the possibility of obtaining their iodo and ethynyl derivatives that may be next subjected to the Sonogashira cross-coupling reaction.

## 2. Results and Discussion

**Scheme 1:**
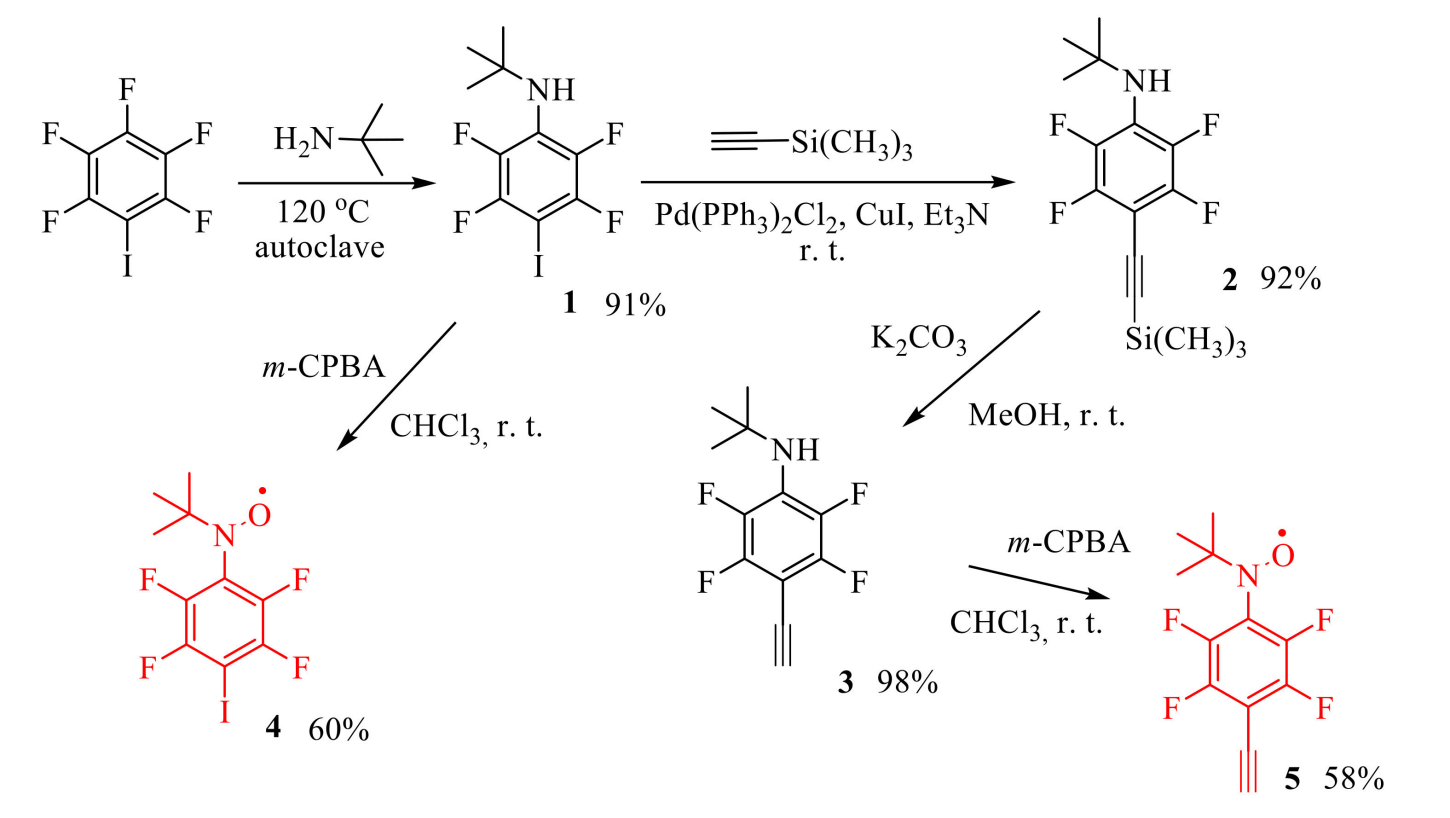
Synthesis of polyfluorinated *tert*-butylarylnitroxides **4** and **5**.

Following the newly developed approach, we carried out the reaction of perfluoroiodobenzene with *tert*-butylamine in chloroform at room temperature (r.t.). As opposed to perfluorotoluene and perfluorobenzonitrile, the iodo derivative did not possess enough reactivity, and the reaction with the amine did not proceed. The use of *tert*-butylamine under autoclaving conditions at 120 °C led to the substitution of the fluorine atom in perfluoroiodobenzene and the formation of *N*-*tert*-butyl-2,3,5,6-tetrafluoro-4-iodoaniline (**1**) in a high yield. Formation of other isomeric amines was not detectable (Supplementary Materials). The oxidation of diamine **1** with *m*-CPBA was performed in CHCl_3_ at r.t. and gave one of the target nitroxide radicals, **4**, in a yield of ~60% (Scheme 1).

4-Iodoaniline **1** was cross-coupled with trimethylsilyl acetylene in dry Et_3_N in the presence of Pd(PPh_3_)_2_Cl_2_ (5 mol%) and CuI (10 mol%) as catalysts. The reaction was carried out at r.t. in a tightly closed Schlenk flask in an argon atmosphere. The process was terminated after the disappearance of ^19^F NMR signals belonging to the starting compound to obtain alkynyl derivative **2** with a yield of ~92%. The resulting compound **2** was cleaved by the action of K_2_CO_3_ in MeOH at r.t., thus producing fluorinated ethynyl-derivative **3** with an almost quantitative yield (Supplementary Materials). At the final stage, amine **3** was oxidized with *m*-CPBA in CHCl_3_ at r.t. and gave ethynyl-substituted nitroxide **5** as a red oily compound in a yield of ~58%. Both nitroxide radicals **4** and **5** were found to be stable and were purified by thin-layer chromatography (TLC).

**Figure 2:**
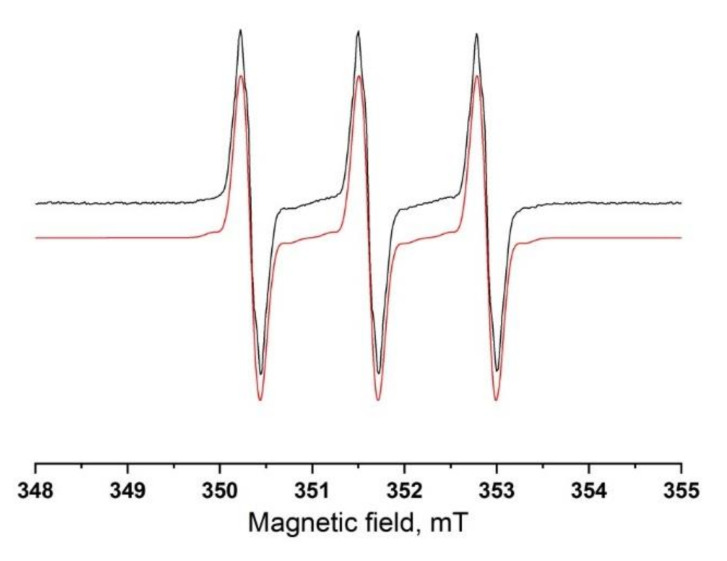
Experimental (black curve) and simulated (red curve) electron spin resonance (ESR) spectra of **4**.

**Figure 3:**
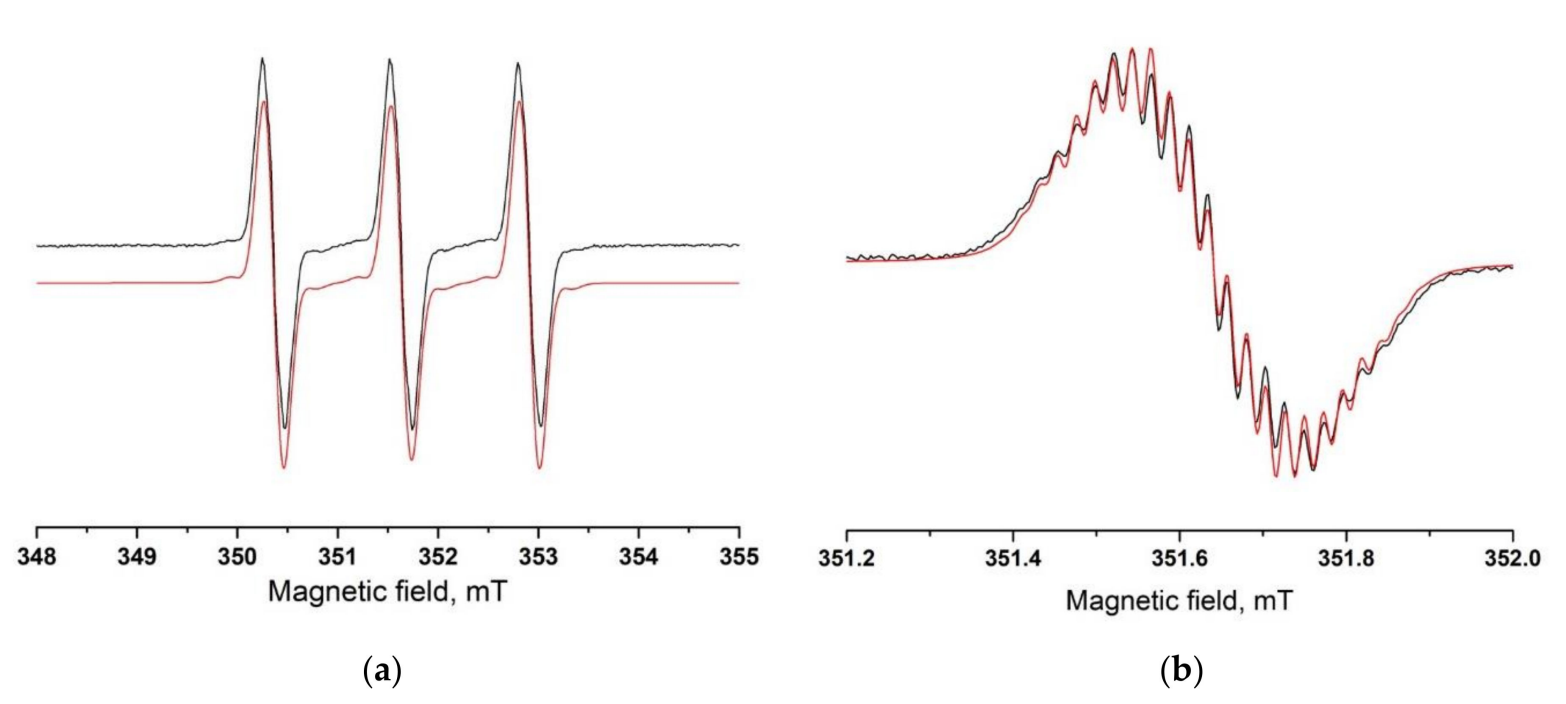
Experimental (black curves) and simulated (red curves) ESR spectra of **5;** (**a**) the whole spectrum; (**b**) the central component.

Electron spin resonance (ESR) spectra (Figure 2 and Figure 3) for diluted (~10^−4^ M) and oxygen-free toluene solutions of radicals **4** and **5** showed triplet patterns at g_iso_ = 2.0065 and g_iso_ = 2.0064, respectively. The spectra were simulated with the following sets of parameters: For **4**, A_N_ = 1.283 mT and A_2F_ = 0.078 mT; for **5**, A_N_ = 1.278 mT. In the case of nitroxide **5**, a high-resolution ESR spectrum was recorded to achieve more complex splitting of the central line of its triplet. The spectrum was well simulated, taking into account nine hyperfine structure (hfs) constants for the protons of the *tert*-butyl group (A_H_ = 0.022 mT), two pairs of hfs constants for the distant fluorine atoms (A_Fortho_ = 0.089 mT; A_Fmeta_ = 0.044 mT) and one hfs constant for the acetylene proton (A_H_ = 0.075 mT).

**Figure 4:**
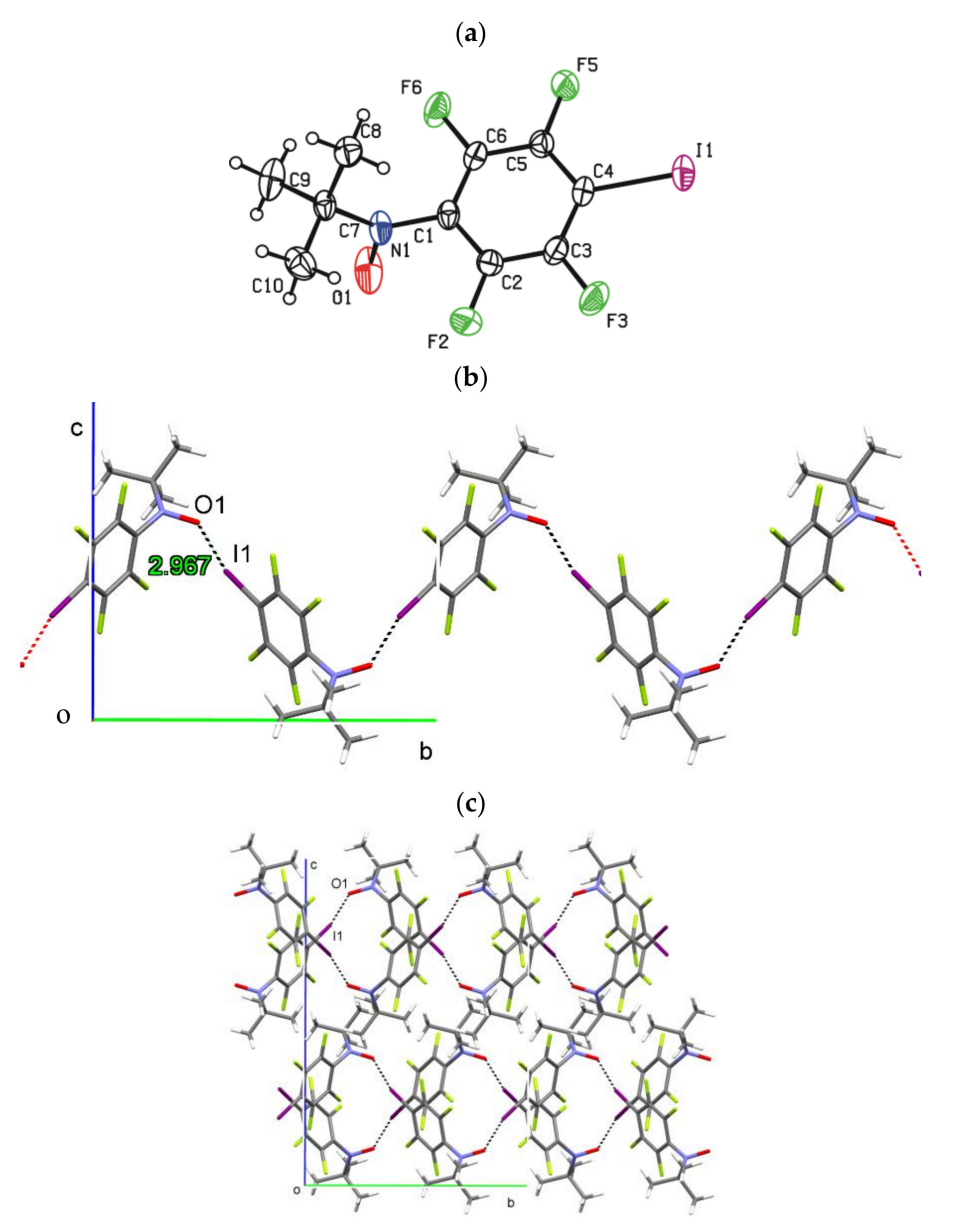
Molecular structure (**a**) and fragments of crystal structure (**b**,**c**) of nitroxide radical **4** (the thermal ellipsoids are drawn at the 30% probability level).

Even though both freshly prepared radicals **4** and **5** were red oils, repeated efforts were made to obtain them in a crystalline form. Eventually, by crystallization from a cold *n*-heptane solution, nitroxide radical **4** was isolated as high-quality crystals that allowed to solve its molecular and crystal structure by X-ray diffraction (XRD) analysis (Figure 4). It was revealed that radical **4** crystallizes in the orthorhombic *Pbca* space group (Table 1). Bond lengths of the *tert*-butylnitroxide moiety (Table 2) are fully consistent with those of previously described radicals of this family [3]. The nitroxide groups in diradical **4** are twisted at a large angle (~64°) relative to the aromatic ring. The observed twisting is due to, first, steric repulsion between the *tert*-butyl group and *ortho*-fluorine atoms and, second, electrostatic repulsion of dipoles C‒F and N‒O. In this regard, the analogous dihedral angle in nonfluorinated *tert*-butylphenylnitroxides is twofold smaller and has experimental and calculated values of 23–34° [9].

The X-ray analysis revealed an assembly of radicals **4** into zigzag chains via halogen bonds between the nitroxide oxygen and iodine atom (Figure 4b,c). If we consider so-called conjugated nitroxides [2], there are very few cases when halogen bonding induces one-dimensional self- or supra-organization of paramagnetic species. Moreover, the halogen bonding with the participation of an iodine atom has been described only for the family of nitronyl nitroxides [10,11,12,13]. For *tert*-butylphenylnitroxides, this type of halogen bonding was shown for the first time. In addition, the halogen bonds observed in nitroxide **4** are very strong, as indicated by the short I1⋯O1 distance (2.967 Å, >15% shorter than the sum of van der Waals radii of O and I [14]) and almost linear C–I⋯O angles (~164°). The N–O (1.278(4) Å) bond distance is typical of nitronyl nitroxide moieties, indicating that the presence of the halogen bond has little impact on electronic properties of the free radicals. This is a consequence of the essentially electrostatic nature of the halogen bond interaction.

An interaction of half an equivalent of bis(hexafluoroacetylacetonato)copper(II) (abbreviated as Cu(hfac)_2_) with radical **4** in CHCl_3_ gave rise to the [Cu(hfac)_2_(**4**)_2_] complex (Supplementary Materials). Its recrystallization from *n*-hexane generated well-shaped crystals suitable for X-ray analysis. If an equivalent amount of Cu(hfac)_2_ was used in the reaction, complex [Cu(hfac)_2_(**4**)_2_] also formed along with the crystallization of excess complex Cu(hfac)_2_. Attempts to prepare a similar complex with nitroxide **5** every time led to the formation of dense intergrowths of crystals of complex [Cu(hfac)_2_(**5**)]. Due to the low quality of the crystals, the structure of the complex was solved with *R*-factor 8.76% and is considered a tentative result here.

**Figure 5:**
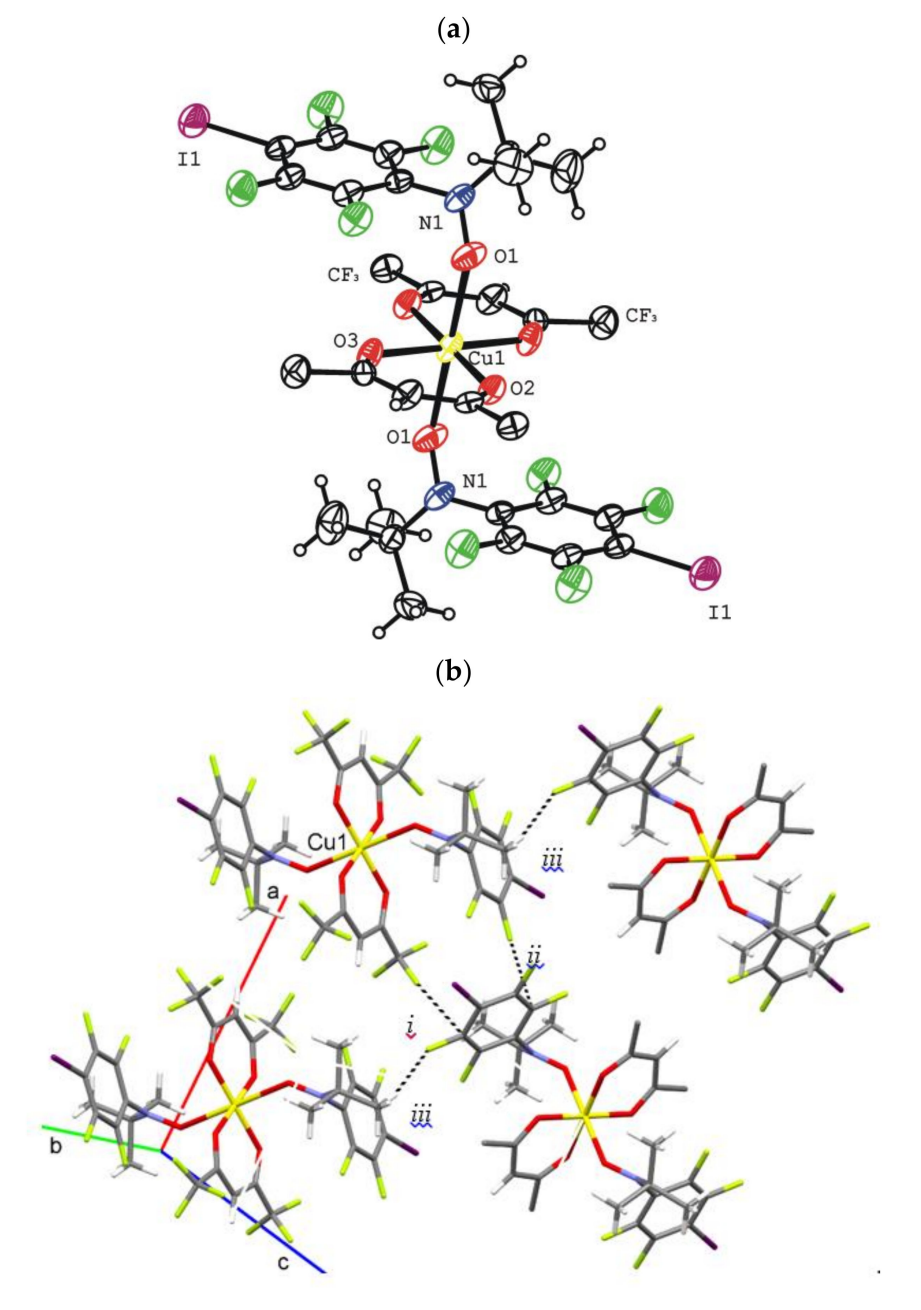
Molecular structure ((**a**) one of the independent molecules) and fragments of crystal structure (**b**) of the [Cu(hfac)_2_(**4**)_2_] complex (the thermal ellipsoids are drawn at the 30% probability level).

According to XRD analysis, coordination compound [Cu(hfac)_2_(**4**)_2_] crystallizes in the triclinic crystal system (space group *P*-1) with two independent molecules per asymmetric unit, as shown in Figure 5a. In the distorted octahedron around the copper ion, two O_NO_ atoms occupy axial positions with *d*_Cu–O_ = 2.380 and 2.400 Å in the independent molecules. Equatorial Cu–O_hfac_ distances in both molecules are in the range of 1.933 to 1.955 Å (Table 2).

In the crystal structure of [Cu(hfac)_2_(**4**)_2_], the independent molecules are bound via two van der Waals interactions of the C–F⋅⋅⋅C_Ph_ type (contacts i and ii). One of the fluorine atoms attached to the aromatic cycle engages in an intermolecular contact (iii) with the H atoms of the *tert*-butyl group, thereby binding the dimers into a layer (Figure 5b).

**Figure 6:**
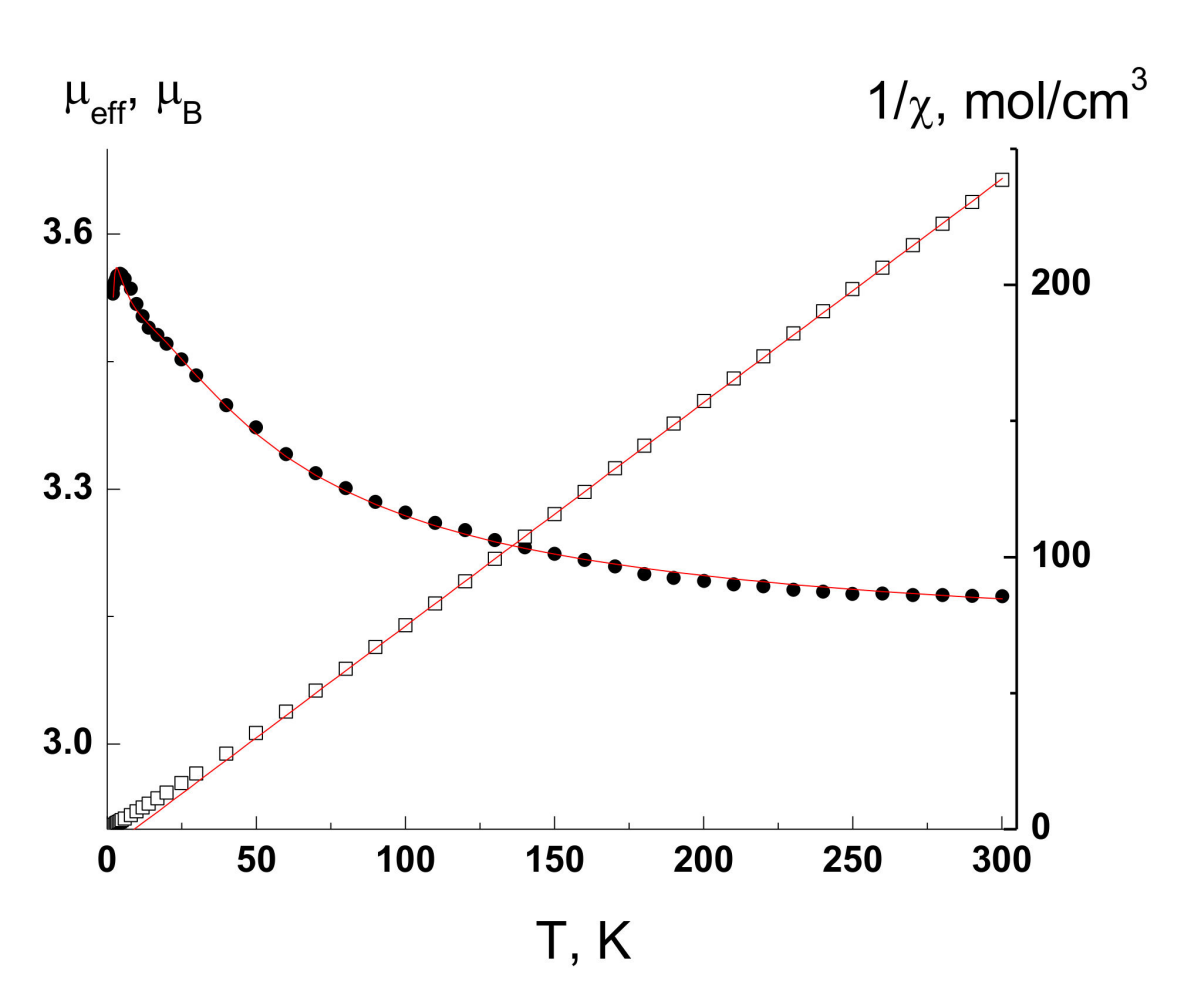
The µ_eff_(*T*) (●) and 1/χ(*T*) (□) dependences for the [Cu(hfac)_2_(**4**)_2_] complex (solid curves are theoretical ones).

Temperature dependences of the effective magnetic moment (µ_eff_) and inverse magnetic susceptibility (1/χ) for complex [Cu(hfac)_2_(**4**)_2_] are shown in Figure 6. The µ_eff_ value is 3.17 µ_B_ at 300 K and increases with decreasing temperature to 3.55 µ_B_ at 4 K. The 1/χ(*T*) dependence is linear in the temperature range 100–300 K and obeys the Curie–Weiss law with best-fit values *C* = 1.218 ± 0.002 K·cm^3^/mol and *θ* = 9.1 ± 0.3 K. The µ_eff_ value at 300 K and Curie constant *C* are in good agreement with theoretical spin-only values of 3.00 µ_B_ and 1.125 K·cm^3^/mol for three paramagnetic centers: Two nitroxides and one Cu(II) ion. The increase in µ_eff_ with decreasing temperature and positive Weiss constant *θ* indicate the domination of ferromagnetic exchange interactions between spins of paramagnetic centers. An analysis of experimental µ_eff_(*T*) dependences was performed via a model of a three-spin exchange cluster (spin Hamiltonian *H* = −2*J*_*Cu*–*R*_ × (*S*_*R*1_*S*_*Cu*_ + *S*_*Cu*_*S*_*R*2_) − 2*J*_*R*–*R*_ × *S*_*R*1_*S*_*R*2_), taking into account intermolecular exchange interactions *zJ′* in the mean field approximation. Best-fit values of g_Cu_ and exchange interaction parameters *J_Cu–R_*, *J_R–R_* and *zJ′* are 2.21, 15.9 cm^−1^, −8.7 cm^−1^ and 0.15 cm^−1^, respectively. g-Factors for the nitroxides were fixed at g_R_ = 2 to avoid overparametrization. The observed ferromagnetic exchange interactions between the unpaired electrons in [Cu(hfac)_2_(**4**)_2_] are consistent with the XRD data on the axial coordination of the nitroxide groups to the Cu^2+^ ion [15,16,17,18].

## 3. Conclusions

The development of the chemistry of nitroxides opens up new areas of their practical application. Recently, we devised a new synthetic approach to functionalized *tert*-butylphenylnitroxides via substitution of a fluorine atom in polyfluorinated arenes by *tert*-butylamine with subsequent oxidation of the obtained *N*-*tert*-butylanilines with *m*-CPBA [3]. With highly activated polyfluorinated compounds, the substitution reaction proceeds smoothly, with the formation of the corresponding aniline. In the case of less activated polyfluorinated aromatics, we proposed the use of lithium *tert*-butylamide, which allowed, for example, to synthesize a necessary diamine precursor for the *N*,*N*′-(perfluorobiphenyl-4,4′-diyl)bis(*N*-*tert*-butyl-*N*-oxyamine) diradical [4]. In the present work, we employed the third version of the reaction, namely, the process was carried out in an autoclave at 120 °C, using *tert*-butylamine both as a reagent and a solvent. Under these conditions, the reaction of perfluoroiodobenzene with *tert*-butylamine proceeded regioselectively and gave *N*-*tert*-butyl-2,3,5,6-tetrafluoro-4-iodoaniline in high yields. The obtained iodo derivative was converted via a multistep procedure into an ethynyl-substituted aryl nitroxide, which was oxidized into the corresponding *tert*-butylphenylnitroxide. The latter radical crystalizes with the formation of the structure in which free radicals are bound into one-dimensional chains by relatively strong halogen bonds N–O···I. If upon the formation of the halogen bond the nitroxide group acts as an acceptor, then in the reaction with Cu(hfac)_2_ it becomes an electron pair donor. The reaction produces a molecular complex of 1:2 composition with axial coordination of the paramagnetic ligands. The result of such coordination gives rise to ferromagnetic intramolecular exchange interactions between Cu(II) and radical spins.

Both synthesized nitroxides, *N*-*tert*-butyl-*N*-oxyamino-2,3,5,6-tetrafluoro-4-iodobenzene and *N*-*tert*-butyl-*N*-oxyamino-2,3,5,6-tetrafluoro-4-ethynylbenzene, represent stable paramagnetic building blocks for the Sonogashira cross-coupling reaction. This reaction is being evaluated for the synthesis of functional paramagnets with potential application in the fields of molecular magnetism and spintronics. In particular, in the course of preliminary experiments, it was shown that the paramagnetic iodo derivative can be successfully used to introduce a spin-bearing group into the planar hexabenzocoronene backbone.

## 4. Materials and Methods

### 4.1. Materials and Measurements

Cu(hfac)_2_ was prepared according to previously described procedures [19]; before use, the Cu(hfac)_2_ complex was sublimated and stored in a desiccator charged with NaOH. The starting materials were obtained from commercial sources (Merck KGaA, Darmstadt, Germany) and were used without further purification. All solvents were purified according to standard procedures. Preparative TLC was performed on Merck precoated silica gel 60 PF254 containing gypsum. The visualization of developed chromatograms was performed by means of UV light.

NMR spectra were recorded on Bruker Avance-300 (300.13 MHz for ^1^H and 282.37 MHz for ^19^F), Avance-400 (400.13 MHz for ^1^H and 100.62 MHz for ^13^C) and DRX-500 (500.13 MHz for ^1^H, 125.76 MHz for ^13^C) spectrometers (Bruker Corporation, Billerica, MA, USA). CDCl_3_ served as a solvent, with residual CHCl_3_ (δ_H_ = 7.26, δ_C_ = 77.0) acting as an internal standard; C_6_F_6_ (δ_F_ = 163.0) was used as an external reference for recording ^19^F NMR spectra. ^13^C NMR spectra were determined with C–H spin decoupling.

Continuous-wave ESR spectra were acquired on a commercial Bruker X Band (9 GHz) spectrometer, Elexsys E 540 (Bruker Corporation, Billerica, MA, USA), at r.t. in dilute (~10^−4^ M) toluene solutions degassed by means of repeated freeze–pump–thaw cycles. ESR spectra were recorded with the following settings: Frequency, 9.87 GHz; microwave power, 2.0 mW; modulation amplitude, 0.01 mT for radical **4** and 0.005 mT for radical **5**; time constant, 20.5 ms; and conversion time, 20 ms. Simulations of solution ESR lines were carried out in the EasySpin software (5.2.28) [20], which is available at http://www.easypin.org.

Masses of molecular ions were determined by high-resolution mass spectrometry (HRMS) on a DFS Thermo Scientific instrument at an ionization energy of 70 eV (Thermo Fisher Scientific, Waltham, MA, USA). Melting points were recorded on a Mettler-Toledo FP81 Thermosystem apparatus (Mettler-Toledo, Greifensee, Switzerland). IR spectra were acquired by means of a Bruker Vector 22 spectrometer in KBr pellets or thin films (Bruker Optik GmbH in Ettlingen). Elemental analyses were performed on a Carlo Erba 1106 CHN elemental analyzer.

Magnetic susceptibility was measured with a MPMSXL SQUID magnetometer (Quantum Design, San Diego, CA) in the temperature range 2 to 300 K with a magnetic field of up to 5 kOe. Diamagnetic corrections were made via Pascal constants. The effective magnetic moment was calculated as follows: μ_eff_(*T*) = ((3k/N_A_μ_B_^2^)χT)^1/2^ ≈ (8χT)^1/2^.

### 4.2. Synthetic Procedures

#### 4.2.1. *N-tert*-Butyl-2,3,5,6-tetrafluoro-4-iodoaniline (**1**)

Pentafluoroiodobenzene (1.50 g, 5.1 mmol) and 2-methylpropan-2-amine (5 mL) were placed into a stainless-steel autoclave (volumetric capacity 50 mL). The reaction mixture was stirred at 120 °C for 90 h. Then, the mixture was allowed to cool down to r.t., placed directly onto a chromatography plate (silica gel) and air-dried. The title product was isolated by TLC with hexane as an eluent (*R_f_* = 0.65) in a 91% yield (1.62 g) as colorless oil. IR (thin), *ν*_max_: 3423, 2968, 2121, 2088, 1801, 1732, 1635, 1487, 1460, 1396, 1369, 1288, 1228, 1203, 1130, 1088, 972, 810 cm^−1^; ^1^H NMR (300 MHz, CDCl_3_): δ = 3.46 (br s, 1H, NH), 1.28 (s, 9H, CH_3_); ^13^C NMR (126 MHz, CDCl_3_): δ = 147.1 [dddd, ^1^*J* (C^3^,F^3^) = 242.3 Hz, ^2^*J* (C^3^,F^2^) = 15.1 Hz, C^3^, C^5^], 140.2 [ddt, ^1^*J* (C^2^,F^2^) = 243.4 Hz, ^2^*J* (C^2^,F^3^) = 16.2 Hz, C^2^, C^6^], 126.6 [tt, ^2^*J* (C^1^,F^2^) = 14.1 Hz, ^3^*J* (C^1^,F^3^) = 2.4 Hz, C^1^], 60.4 [t, ^2^*J* (C^4^,F^3^) = ^2^*J* (C^4^,F^5^) = 28.1 Hz, C^4^], 54.5 (s, C^7^), 30.1 [t, *J* (C^8^,F^2^) = *J* (C^8^,F^6^) = 2.7 Hz, C^8^]; ^19^F NMR (282 MHz, CDCl_3_): δ = −124.1 (m, 2F, F^3^, F^5^), −149.9 (m, 2F, F^2^, F^6^); HRMS (EI): *m*/*z* [M]^+^ calcd. for C_10_H_10_F_4_NI: 346.9789; found 346.9790.

#### 4.2.2. *N-tert*-Butyl-2,3,5,6-tetrafluoro-4-((trimethylsilyl)ethynyl)aniline (**2**)

Pd(PPh_3_)_2_Cl_2_ (50 mg, 0.07 mmol) and CuI (27 mg, 0.144 mmol) were added to a stirred solution of iodoaryl **1** (500 mg, 1.44 mmol) and ethynyltrimethylsilane (282 mg, 2.88 mmol) in dry Et_3_N (20 mL) at r.t. in an argon atmosphere. The mixture was stirred at r.t. for 24 h, placed directly onto a chromatographic plate (silica gel) and air-dried. The title product was isolated by TLC with hexane as an eluent (*R_f_* = 0.80) in a 92% yield (420 mg) as colorless oil. IR (thin), *ν*_max_: 3427, 2966, 2160, 1651, 1500, 1471, 1435, 1252, 1230, 1203, 983, 847, 762, 702, 677 cm^−1^; ^1^H NMR (300 MHz, CDCl_3_): δ = 3.34 (br s, 1H, NH), 1.29 [s, 9H, C(CH_3_)_3_], 0.24 [s, 9H, Si(CH_3_)_3_]; ^13^C NMR (100 MHz, CDCl_3_): δ = 147.5 [dddd, ^1^*J* (C^3^,F^3^) = 249.7 Hz, ^2^*J* (C^3^,F^2^) = 14.6 Hz, C^3^, C^5^], 139.2 [ddt, ^1^*J* (C^2^,F^2^) = 239.3 Hz, ^2^*J* (C^2^,F^3^) = 14.6 Hz, C^2^, C^6^], 127.1 [tt, ^2^*J* (C^1^,F^2^) = 13.7 Hz, ^3^*J* (C^1^,F^3^) = 2.6 Hz, C^1^], 105.7 [t, *J* (C^7^,F^3^) = *J* (C^7^,F^5^) = 3.4 Hz, C^7^], 94.4 [tt, ^2^*J* (C^4^,F^3^) = ^2^*J* (C^4^,F^5^) = 18.4 Hz, C^4^], 89.4 [t, *J* (C^8^,F^5^) = *J* (C^8^,F^3^) = 3.9 Hz, C^8^], 54.3 (s, C^10^), 30.2 [t, *J* (C^11^,F^2^) = *J* (C^11^,F^6^) = 3.0 Hz, C^11^], −0.4 (s, C^9^); ^19^F NMR (282 MHz, CDCl_3_): δ = −139.9 (m, 2F, F^3^, F^5^), −154.2 (m, 2F, F^2^, F^6^); HRMS (EI): *m*/*z* [M]^+^ calcd. for C_15_H_19_F_4_NSi: 317.1217; found 317.1215.

#### 4.2.3. *N-tert*-Butyl-4-ethynyl-2,3,5,6-tetrafluoroaniline (**3**)

To a solution of **2** (230 mg, 0.72 mmol) in MeOH (10 mL), K_2_CO_3_ (200 mg, 1.45 mmol) was added. The reaction mixture was stirred at r.t. for 3.5 h, then placed directly onto a chromatographic plate (silica gel) and air-dried. The title product was isolated by TLC with hexane as an eluent (*R_f_* = 0.50) in a 98% yield (174 mg) as colorless oil. IR (thin), *ν*_max_: 3426, 3307, 2974, 2875, 2117, 1705, 1651, 1500, 1471, 1433, 1471, 1369, 1303, 1232, 1203, 1172, 1041, 985, 974, 893, 806, 673, 605, 432 cm^−1^; ^1^H NMR (300 MHz, CDCl_3_): δ = 3.63 (br s, 1H, NH), 3.45 (s, 1H, H^8^), 1.30 (t, 9H, C(CH_3_)_3_); ^13^C NMR (126 MHz, CDCl_3_): δ = 147.9 [dddd, ^1^*J* (C^3^,F^3^) = 250.2 Hz, ^2^*J* (C^3^,F^2^) = 14.4 Hz, C^3^, C^5^], 139.0 [ddt, ^1^*J* (C^2^,F^2^) = 239.1 Hz, ^2^*J* (C^2^,F^3^) = 14.5 Hz, C^2^, C^6^], 127.7 [tt, ^2^*J* (C^1^,F^2^) = 13.5 Hz, ^3^*J* (C^1^,F^3^) = 2.7 Hz, C^1^], 92.7 [tt, ^2^*J* (C^4^,F^3^) = ^2^*J* (C^4^,F^5^) = 18.2 Hz, C^4^], 87.0 [t, *J* (C^8^,F^5^) = *J* (C^8^,F^3^) = 3.4 Hz, C^8^], 69.4 [t, *J* (C^7^,F^3^) = *J* (C^7^,F^5^) = 4.0 Hz, C^7^], 54.2 (s, C^9^), 30.3 [t, *J* (C^11^,F^2^) = *J* (C^11^,F^6^) = 3.1 Hz, C^10^]; ^19^F NMR (282 MHz, CDCl_3_): δ = −140.1 (m, 2F, F^3^, F^5^), −154.2 (m, 2F, F^2^, F^6^); HRMS (EI): *m*/*z* [M]^+^ calcd. for C_12_H_11_F_4_N: 245.0822; found 245.0823.

#### 4.2.4. *N-tert*-Butyl-*N*-oxyamino-2,3,5,6-tetrafluoro-4-iodobenzene (**4**)

A solution of *N*-*tert*-butyl-2,3,5,6-tetrafluoro-4-iodoaniline **1** (0.7 mmol) and *m*-CPBA (1.0 mmol, 172 mg) in CHCl_3_ (5 mL) was stirred at r.t. for 24 h. Column chromatography (SiO_2_ or Al_2_O_3_ column 3 × 20 cm, CHCl_3_ as an eluent) afforded a red fraction of radical **4**. The solvent was removed under reduced pressure at r.t. to obtain title compound **4**. Red crystals; yield 60% (150 mg); mp 89.8 °C (decomp.). IR (KBr), *ν*_max_: 2981, 1626, 1483, 1462, 1398, 1350, 1275, 1248, 1196, 970, 800, 773, 721, 579 cm^−1^; UV-Vis (EtOH) λ_max_/nm (lg ε): 235 (3.16), 307 (2.60); HRMS (EI): *m*/*z* [M]^+^ calcd. for C_10_H_9_F_4_NOI: 361.9660; found 361.9662; Anal. calcd. for C_10_H_9_F_4_NOI: C, 33.17; H, 2.51; N, 3.87. Found: C, 33.44; H, 2.43; N, 3.87.

#### 4.2.5. *N-tert*-Butyl-*N*-oxyamino-2,3,5,6-tetrafluoro-4-ethynylbenzene (**5**)

The compound was obtained through the oxidation of amine **3** in a manner similar to the preparation of nitroxyl **4**. Red oil; yield 58% (105 mg). IR (thin), *ν*_max_: 3305, 3257, 2983, 2943, 2123, 1722, 1680, 1643, 1616, 1491, 1396, 1367, 1304, 1252, 1201, 989, 835, 787, 769, 735, 677, 642, 584, 501 cm^−1^; UV-Vis (EtOH) λ_max_/nm (lg ε): 239 (3.11), 249 (3.08), 317 (2.41); HRMS (EI): *m*/*z* [M]^+^ calcd. for C_12_H_10_F_4_NO: 260.0693; found 260.0691.

#### 4.2.6. *trans*-Bis(1,1,1,5,5,5-hexafluoropentane-2,4-dionato-*κ*^2^*O*,*O*′)bis{*N*-*tert*-butyl-*N*-oxyamino-2,3,5,6-tetrafluoro-4-iodobenzene-*κO*}copper(II) ([Cu(hfac)_2_(**4**)_2_])

Cu(hfac)_2_·H_2_O (248 mg, 0.5 mmol) was added to a solution of radical **4** (362 mg, 1.0 mmol) in CHCl_3_ (10 mL). The reaction mixture was stirred for 30 min and then was incubated at −15 °C for 10 h. The solution was filtered and evaporated. The residue was dissolved in *n*-hexane (5 mL) and the resultant solution was filtered and incubated at −15 °C for 10 h to prepare crystals that were filtered off and air-dried. Brown crystals; yield 78%; IR (KBr) $\tilde{v}$_max_, cm^−1^: 3435, 3001, 2985, 1641, 1597, 1558, 1527, 1485, 1400, 1354, 1261, 1207, 1149, 1107, 1030, 968, 816, 798, 773, 746, 723, 681, 596, 581, 530; calcd. for C_30_H_20_N_2_CuF_20_I_2_O_6_: C 29.98, H 1.68, F, 31.62; N 2.33; found C 30.30, H 1.57, F 31.76, N 2.07.

### 4.3. Single-Crystal XRD Analyses

XRD data were collected at r.t. on a Bruker Kappa Apex II CCD diffractometer with Mo Kα radiation (λ = 0.71073 Å) and a graphite monochromator (Table 1). Absorption corrections were applied empirically using *SADABS* programs [21]. The structures were solved by direct methods by means of the SHELXS-97 software suite [22,23] and were refined using the full-matrix least-squares method against all *F*^2^ in anisotropic approximation (beside the H atoms) using the SHELXL2014/7 software suite [24]. The H atoms’ positions were calculated with the riding model. The asymmetric unit of [Cu(hfac)_2_(**4**)_2_] contains halves of two independent moieties.

CCDC 2038555, 2,038,556 and 2,039,515 (preliminary data) contain the crystallographic data for **4**, [Cu(hfac)_2_(**4**)_2_] and [Cu(hfac)_2_(**5**)]. These data can be obtained free of charge via http://www.ccdc.cam.ac.uk/cgi-bin/catreq.cgi or from the Cambridge Crystallographic Data Centre, 12 Union Road, Cambridge CB2 1EZ, UK; fax: (+44) 1223 336 033; or e-mail: deposit@ccdc.cam.ac.uk.

## Figures and Tables

**Table 1:** XRD data for compounds **4**, [Cu(hfac)_2_(**4**)_2_] and [Cu(hfac)_2_(**5**)].

Compound	4	[Cu(hfac)_2_(4)_2_]	[Cu(hfac)_2_(5)]
Empirical formula	C_10_H_9_F_4_INO	C_30_H_20_CuF_20_I_2_N_2_O_6_	C_22_H_12_CuF_16_NO_5_
Formula weight	362.08	1201.82	737.87
Crystal system	Orthorhombic	Triclinic	Triclinic
Space group	*Pbca*	*P*¯1	*P*¯1
Unit cell dimensions *a*, Å	11.5462(4)	10.0763(9)	9.132(1)
*b*, Å	12.1680(5)	13.784(1)	10.472(1)
*c*, Å	17.8601(7)	16.009(2)	17.273 (2)
*α*, °	90	96.469(3)	75.370(6)
*β*, °	90	102.396(4)	83.253(6)
*γ*, °	90	104.941(3)	65.505(5)
Volume, Å^3^	2509.2(2)	2064.7(3)	1454.3(3)
*Z*	8	2	2
*F*(000)	1384	1154	728
Density (calcd.), Mg·m^−3^	1.917	1.933	1.685
Abs. coefficient, mm^−1^	2.59	2.16	0.89
Crystal size, mm	0.90 × 0.30 × 0.10	0.30 × 0.15 × 0.01	0.25 × 0.24 × 0.14
*T*_min_, *T*_max_	0.544, 0.862	0.722, 0.862	0.825, 0.928
Reflections collected	28215	34134	28365
Independent reflections [*R*_int_]	3516	7322	5688
Reflections observed (I > 2σ(I))	2741	3850	2856
*R*_int_	0.048	0.089	0.074
θ range for data collection (°)	2.3–30.4	1.6–25.2	2.194–26.088
Range of *h*,	−13→16	−12→11	−11→11
*k*,	−16→16	−16→16	−12→12
*l*	−23→24	−19→18	−21→21
Completeness to θ 50° (%)	100	99.1	99.7
Data/restraints/parameters	3516/0/157	7322/0/553	5688/6/503
Final *R* [*F*^2^ > 2 σ (*F*^2^)]	0.040	0.062	0.087
Final *wR*(*F*^2^) (all data)	0.115	0.173	0.324
Goodness-of-fit on *F*^2^	1.03	1.01	1.05
Largest diff. peak/hole, e·Å^−3^	1.44, −1.12	0.71, −0.64	0.64, −0.56

**Table 2:** Selected geometrical parameters for **4** and [Cu(hfac)_2_(**4**)_2_] (two independent molecules).

Compound	4	[Cu(hfac)_2_(4)_2_]	[Cu(hfac)_2_(4)_2_]′
Bond lengths, Å			
Cu1–O1	-	2.401(8)	2.380(7)
Cu1–O2	-	1.933(6)	1.954(6)
Cu1–O3	-	1.955(6)	1.954(5)
C4–I1	2.079(4)	2.062(9)	2.060(8)
N1–O1	1.278(4)	1.29(1)	1.283(9)
N1–C1	1.428(4)	1.43(1)	1.41(1)
N1–C7	1.493(5)	1.47(1)	1.50(1)
Bond angles, °			
O2–Cu1–O1	-	95.7(2)	98.6(2)
O3–Cu1–O1	-	91.5(3)	91.2(2)
O2–Cu1–O3	-	92.3(2)	92.3(2)
O1–N1–C1	115.7(3)	115.8(7)	115.9(6)
O1–N1–C7	118.0(3)	117.2(7)	116.8(6)
C1–N1–C7	125.3(3)	125.7(7)	126.4(6)

## Supplementary Materials

The Supplementary Materials are available online. NMR spectra and the IR spectrum of [Cu(hfac)_2_(**4**)_2_].
