# Supplementary material for: Fluorinated Organic Paramagnetic Building Blocks for Cross-Coupling Reactions
Source: Molecules. 2020 Nov 19;25(22):5427. doi: 10.3390/molecules25225427 (PMC7699513; doi:10.3390/molecules25225427)
Supplement: Supplementary file 1 [file molecules-25-05427-s001.pdf]

Supplementary Materials

Fluorinated Organic Paramagnetic Building Blocks for Cross-Coupling Reactions

Larisa V. Politanskaya [1], Pavel A. Fedyushin [1], Tatyana V. Rybalova [1], Artem S. Bogomyakov [2], Nargiz B. Asanbaeva [1] and Evgeny V. Tretyakov [1,3,*]

[1] N.N. Vorozhtsov Novosibirsk Institute of Organic Chemistry, 9 Ac. Lavrentiev Avenue, Novosibirsk 630090, Russia; plv@nioch.nsc.ri (L.P.); feduyshin@nioch.nsc.ru (P.F.); rybalova@nioch.nsc.ru (T.R.); nasanbaeva@nioch.nsc.ru (N.A.)

[2] International Tomography Center, Siberian Branch of Russian Academy of Sciences, 3a Institutskaya Str., Novosibirsk 630090, Russia; bus@tomo.nsc.ru (A.B.)

[3] N.D. Zelinsky Institute of Organic Chemistry, Leninsky Prospect, 47, Moscow 119991, Russia

* Correspondence: tretyakov@nioch.nsc.ru (E.T.)

Table of contents:

NMR spectra……………………………………………………………………………S2

IR spectrum of ([Cu(hfac)$_2$(**4**)$_2$]……………………………..…………………………S7

^{1}H NMR spectra ($CDCl_3$, residual $CHCl_3$ δ_H = 7.26 ppm) Bruker Avance-300 (300.13 MHz) of **1**

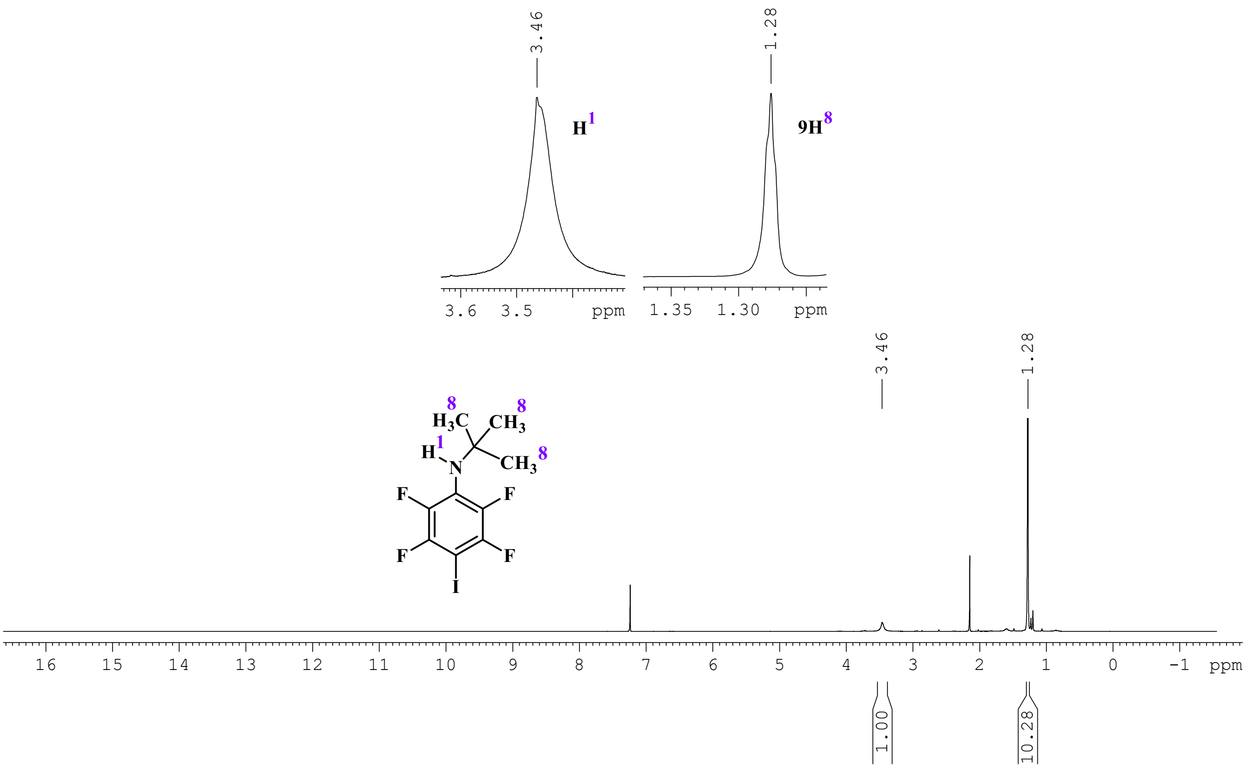

^{19}F NMR spectra ($CDCl_3$, C_6F_6 δ_F = 0.0 ppm) (Bruker Avance-300 (282.37 MHz) of **1**

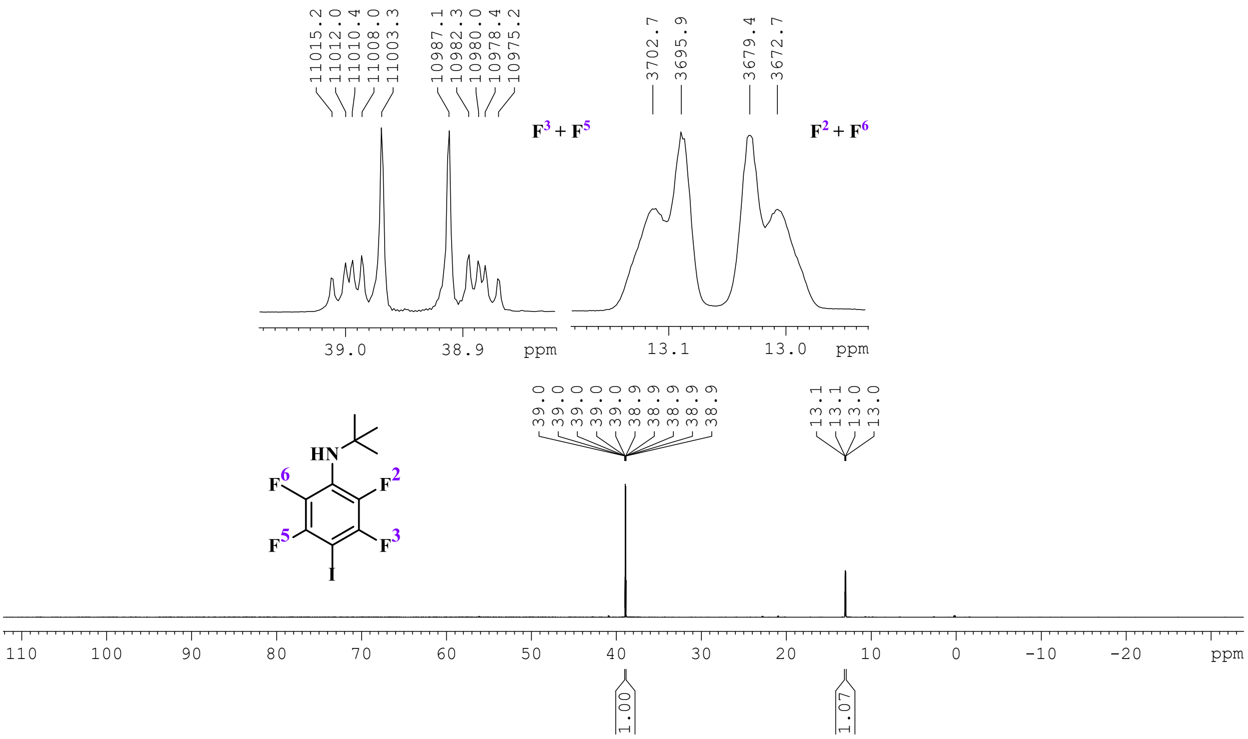

^{13}C NMR spectra ($CDCl_3$, δ_c = 77.0 ppm) Bruker DRX-500 (126 MHz) of **1**

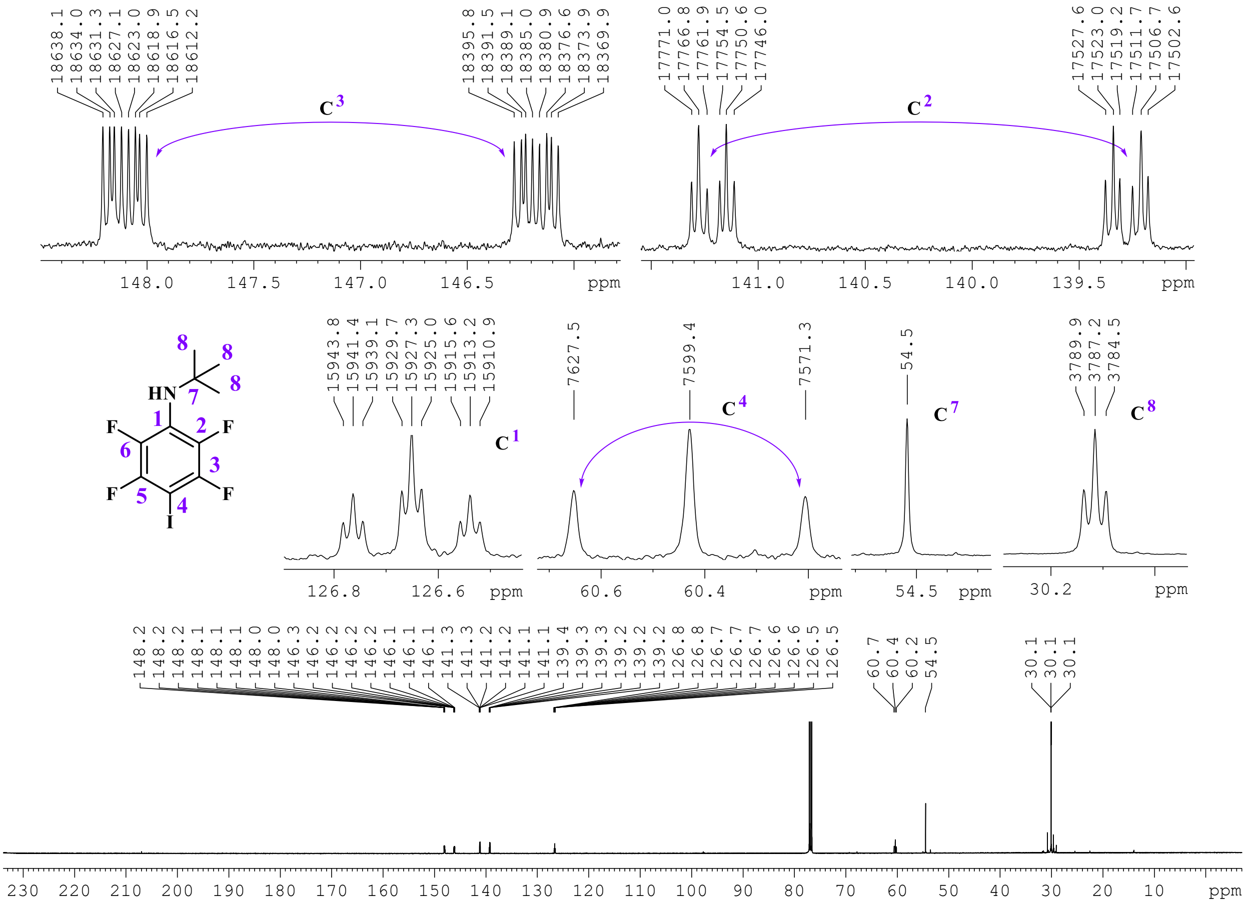

^{1}H NMR spectra ($CDCl_3$, residual $CHCl_3$ δ_H = 7.26 ppm) Bruker Avance-300 (300.13 MHz) of **2**

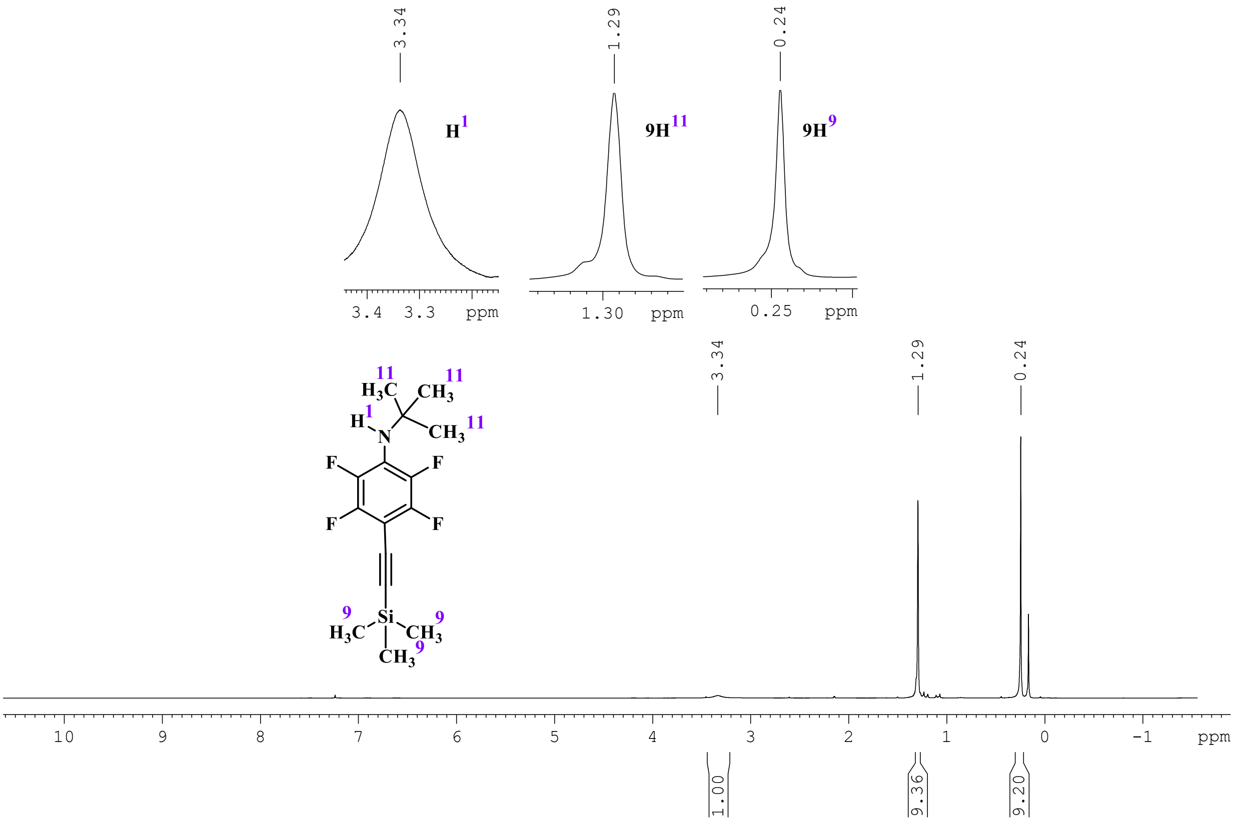

^{19}F NMR spectra ($CDCl_3$, C_6F_6 δ_F = 0.0 ppm) Bruker Avance-300 (282.37 MHz) of **2**

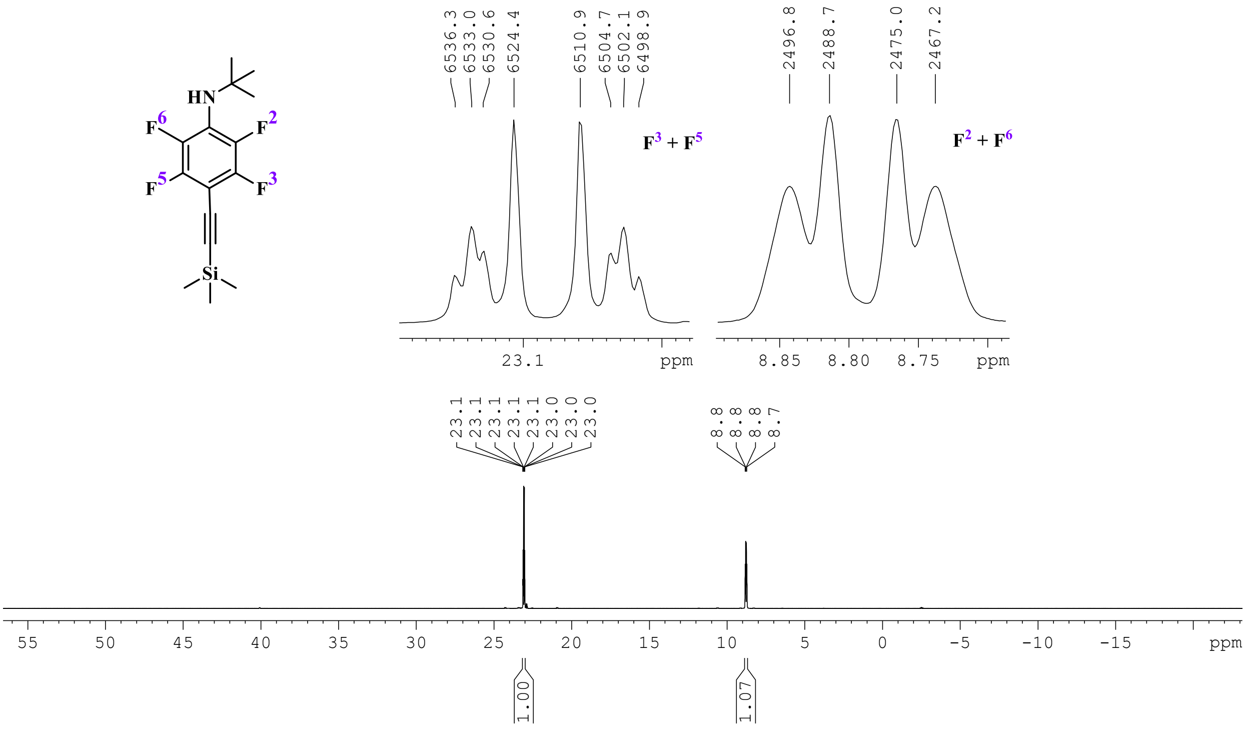

[13] C NMR spectra ($CDCl_3$, δ_c = 77.0 ppm) Bruker Avance-400 (100.62 MHz) of **2**

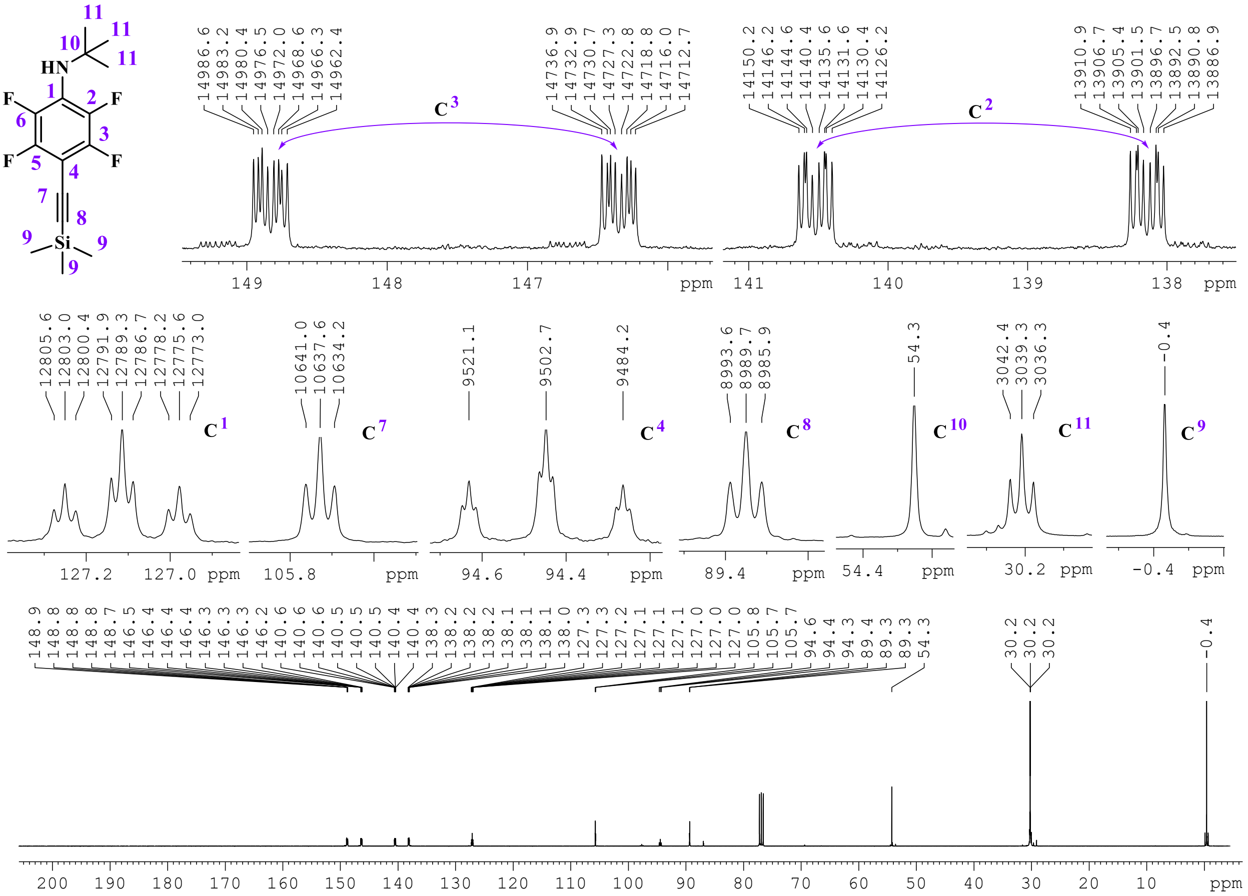

[1] H NMR spectra ($CDCl_3$, residual $CHCl_3$ δ_H = 7.26 ppm) Bruker Avance-300 (300.13 MHz) of **3**

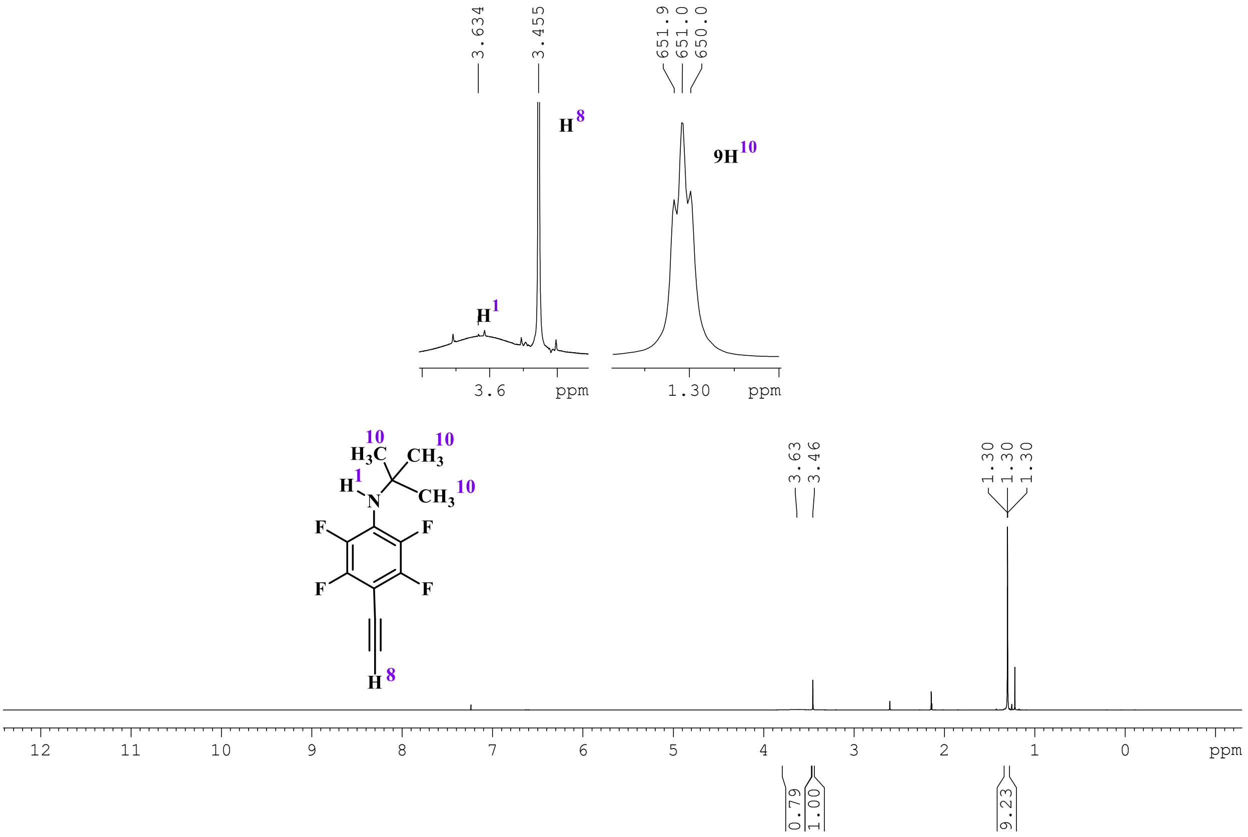

^{19}F NMR spectra ($CDCl_3$, C_6F_6 δ_F = 0.0 ppm) Bruker Avance-300 (282.37 MHz) of **3**

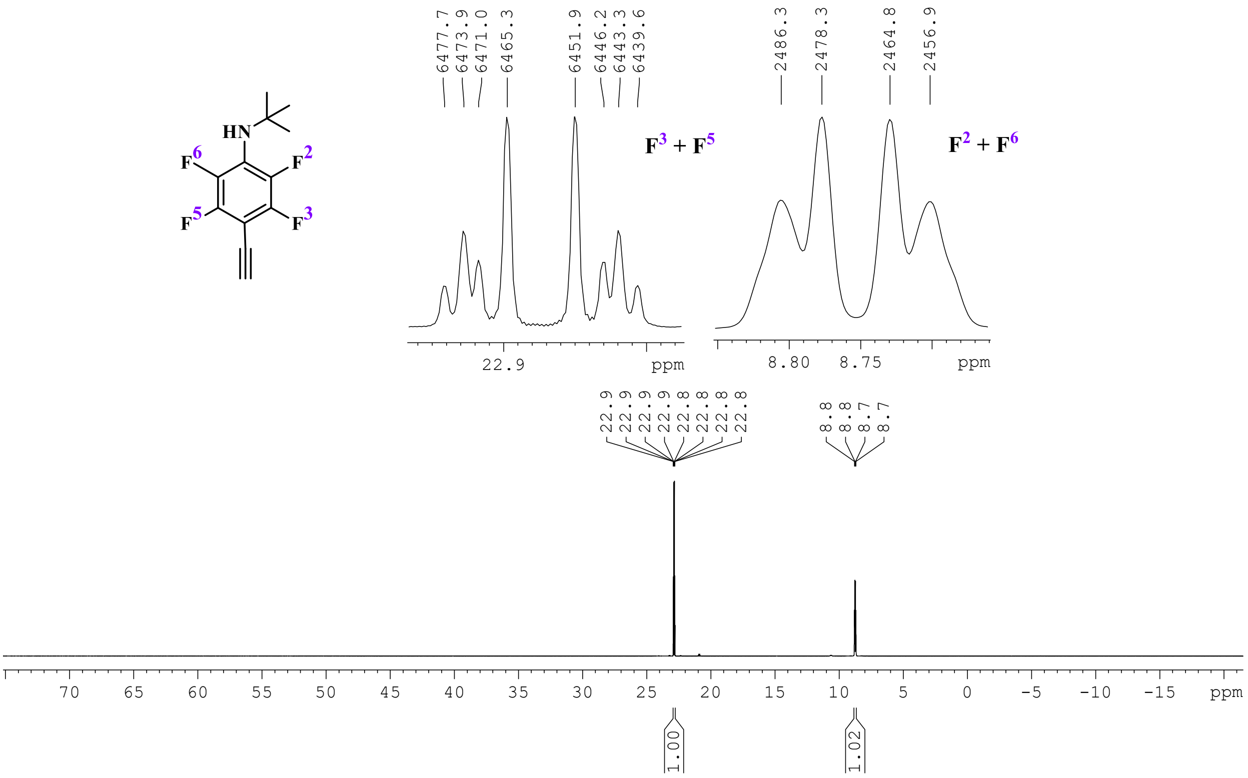

^{13}C NMR spectra ($CDCl_3$, δ_c = 77.0 ppm) Bruker DRX-500 (126 MHz) of **3**

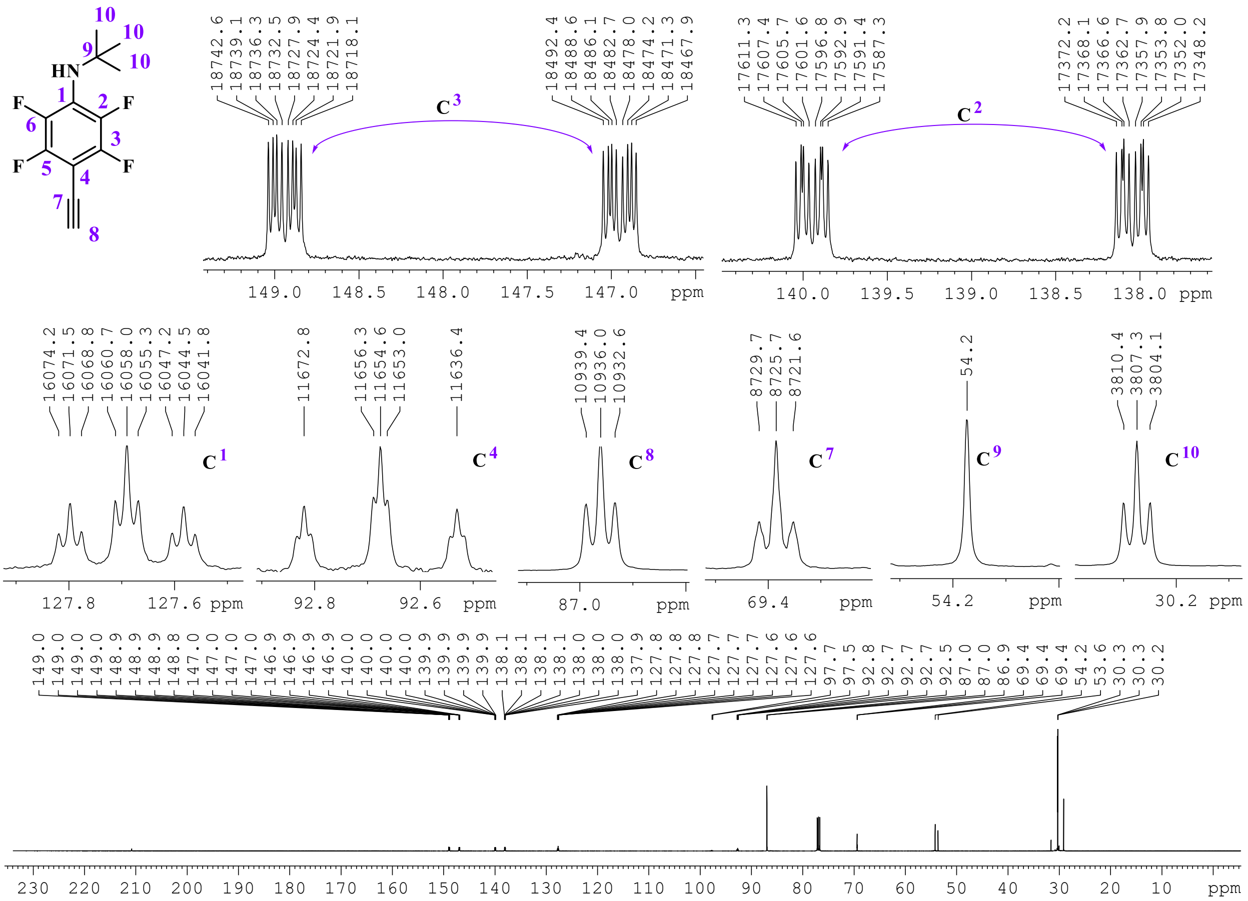

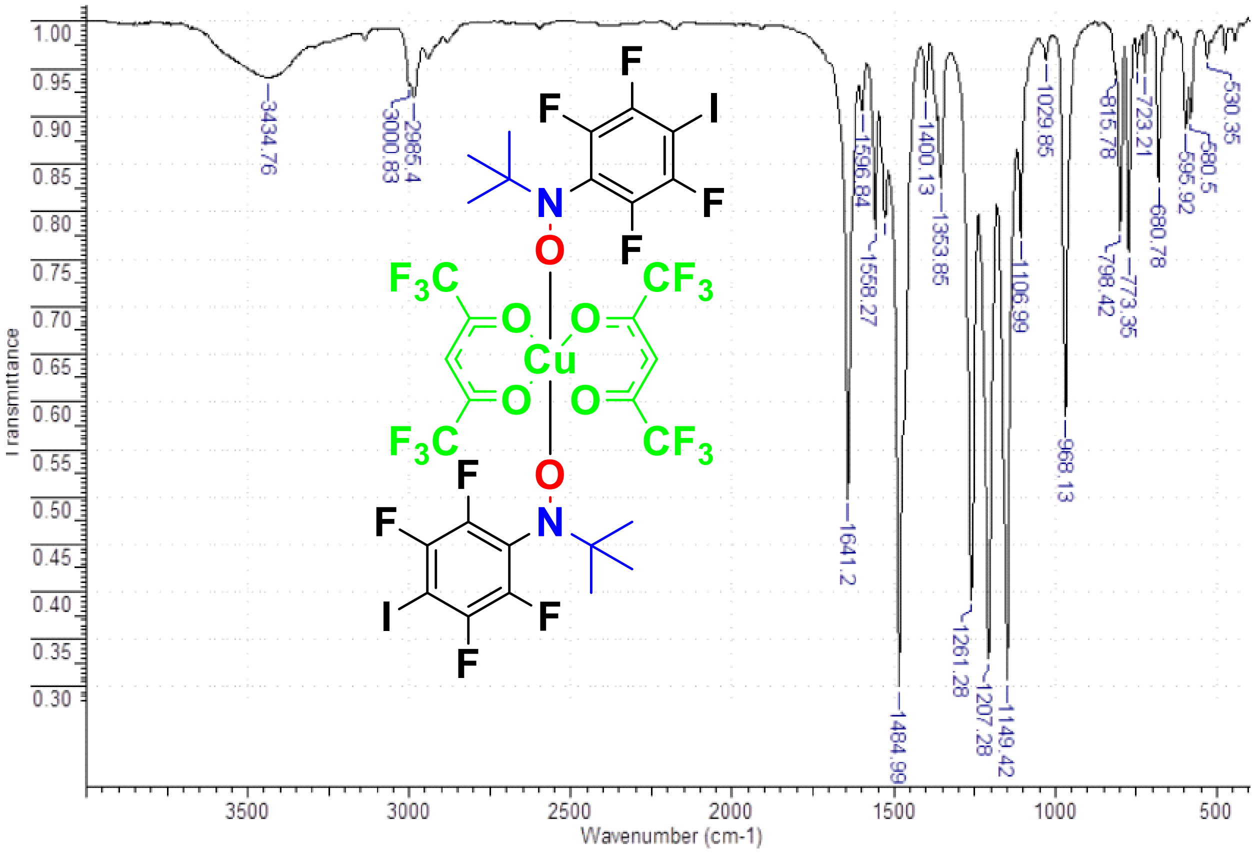
Transmittance
Wavenumber (cm-1)
3434.76
3000.83
2985.4
1641.2
1596.84
1558.27
1484.99
1400.13
1353.85
1261.28
1207.28
1149.42
1106.99
1029.85
968.13
815.78
798.42
773.35
723.21
680.78
595.92
580.5
530.35
Cu
